# Overexpression of DRAM enhances p53-dependent apoptosis

**DOI:** 10.1002/cam4.39

**Published:** 2013-02-03

**Authors:** Masahiro Takahashi, Yuichi Kakudo, Shin Takahashi, Yasuhiro Sakamoto, Shunsuke Kato, Chikashi Ishioka

**Affiliations:** Department of Clinical Oncology, Institute of Development, Aging and Cancer, Tohoku University, 4-1 Seiryo-machi, Aoba-ku, Sendai, Japan

**Keywords:** Apoptosis, DRAM, p53, super p53

## Abstract

Tumor suppressor p53-dependent apoptosis is thought to be one of the most important tumor-suppressive mechanisms in human tumorigenesis. Till date, “super p53” mutants exhibiting more potent ability to induce apoptosis than wild-type p53 have been reported. These super p53s may provide a clue for development of novel therapeutic targets. However, the major mechanism underlying the super p53-dependent apoptosis remains unclear. To identify critical gene(s) in this mechanism, we performed a comprehensive and comparative expression analysis in p53-null Saos-2 cells with conditional expression of wild-type p53 and S121F, which was previously reported as a super p53 mutant. We identified damage-regulated autophagy modulator (*DRAM*) as one of the genes that were more upregulated by S121F than wild-type p53. Although knockdown of DRAM was not sufficient for reducing the ability of S121F to induce apoptosis, DRAM overexpression enhanced the ability in a wild-type p53-dependent manner. Here, we show that *DRAM* is an important gene for the enhancement of p53-dependent apoptosis. Additional analysis of the mechanism of super p53-dependent apoptosis may lead to the identification of novel drug targets for cancer therapy.

## Introduction

Tumor suppressor p53 protein is activated by various cellular stresses, including DNA damage and hypoxia, and is posttranslationally phosphorylated and acetylated. Activated p53 binds to specific DNA sequences in the regulatory regions of downstream genes, resulting in cellular events such as cell cycle arrest and apoptosis [1–4]. Thus far, several downstream genes have been isolated that are involved in cell cycle regulation [5–8], apoptosis [9–20], DNA repair [21,22], angiogenesis [23,24], and p53 stability regulation [25]. Hence, loss of p53 function fails to activate these genes after cellular stress and is thus thought to be a critical cause of tumorigenesis and/or tumor progression [26].

p53-dependent apoptosis is one of the most critical pathways of cell death in tumor-suppressive functions. Apoptosis is an evolutionarily conserved programmed cell death process. So far, numerous studies have reported results from protein investigations on p53-dependent apoptotic mechanisms. A number of p53-target gene products, such as BAX, PUMA, NOXA, p53AIP1, and PTEN, elicit apoptosis. Apart from the known transactivation-dependent mechanism, a transactivation-independent mechanism via mitochondria has also been reported [27]. Eventually, p53-dependent apoptosis is induced by release of caspases that directly execute apoptosis.

Previous studies of p53 mutants have revealed the presence of “super p53” mutants that exhibit increased ability to induce apoptosis than wild-type p53 [28,29]. Because the analysis of the mechanism of super p53-dependent apoptosis may reveal an innovative drug development target and a new site of action, these super p53s are likely to be extremely useful for cancer therapy. Kakudo et al. evaluated several super p53s for their ability to induce apoptosis in Saos-2 cells and their sequence-specific transcriptional activities on six p53-target genes, but their study did not elucidate the essential mechanisms responsible for the increased ability to induce apoptosis [29].

We first established Saos-2 cell lines that contained doxycycline (Dox)-inducible wild-type p53, S121F (a known super p53 mutant), or R175H (a known common mutant), and performed microarray analysis to identify an important candidate gene for the strong ability of S121F to induce apoptosis. In this study, we show that damage-regulated autophagy modulator (*DRAM*) is one of the genes that are more strongly induced by S121F than wild-type p53. DRAM has been reported as a direct target of p53 and a critical factor for p53-dependent apoptosis and autophagy in specific contexts [30]. Furthermore, we show that DRAM enhances the ability to induce apoptosis in a wild-type p53-dependent manner and potentially contributes to the enhanced ability of S121F to induce apoptosis.

## Materials and Methods

### Cell culture

Human cell lines Saos-2 (osteosarcoma, *TP53*^*−/−*^) and SF126 (glioblastoma, *TP53*^*−/−*^) were cultured in RPMI 1640 containing 10% fetal bovine serum at 37°C. p53-inducible cell lines were cultured in a medium containing 200 μg/mL hygromycin B (Life Technologies, Carlsbad, CA).

### Plasmid construction and transfection

The wild-type p53 expression vector, pCR259-p53-WT, and the S121F mutant p53 expression vector, pCR259-p53-S121F, were constructed as described previously [29]. A FLAG-tagged DRAM expression vector was constructed by inserting *DRAM* cDNA into the *Eco*RI/*Bam*I site of the p3XFLAG-CMV-14 vector (Sigma-Aldrich, St. Louis, MO). The polymerase chain reaction (PCR) primers for the *DRAM* cDNA were 5′-GTAGAATTCATGCTGTGCTTCCTGAGGGGAAT-3′ and 5′-GCCGGATCCAATATACCATTGATTTCTGTGG-3′. The *DRAM* cDNA was sequenced using an automated CEQ2000XL DNA analysis system (Beckman Coulter, Brea, CA). These expression vectors were transfected using Effectene transfection reagent (Qiagen, Valencia, CA).

### p53-inducible cell lines

p53-inducible cell lines were established as described previously [20]. Wild-type or mutant p53s were induced with 10 ng/mL Dox (Sigma-Aldrich) in the established clones, Saos-2Tet-p53-WT, -S121F, and -R175H or SF126Tet-p53-WT, -S121F, and -R175H.

### Oligonucleotide microarray analysis

Saos-2-Mock, Saos-2Tet-p53-WT, -S121F, and -R175H cells were grown to 70–80% confluence in 6-cm dishes and further incubated with or without Dox for 24 h. Total RNA was extracted using the RNeasy Mini kit (Qiagen). Total RNA (800 ng) from each sample was used for the microarray analysis. The quality of total RNA was assessed using the 2100 Bioanalyzer (Agilent Technologies, Santa Clara, CA). Comprehensive and comparative mRNA expression analysis was performed using the Whole Human Genome microarray (Agilent Technologies) according to the manufacturer's protocol. The gene expression data were analyzed using Gene Spring v9.2 (Agilent Technologies) and TIGR open access software TMEV v4.0. The results are shown using TMEV v4.0.

### Real-time quantitative RT-PCR

Total RNA was extracted using the RNeasy Mini kit 24 h after Dox treatment. cDNA was generated from 2 μg of total RNA using the High Capacity cDNA Reverse Transcription kit (Life Technologies). Real-time quantitative RT-PCR (qRT-PCR) was performed in duplicate using TaqMan Gene Expression Assays (Life Technologies), and reactions were analyzed using the CFX96 thermal cycler (Bio-Rad, Hercules, CA). *DRAM* mRNA levels were normalized to *ACTB* mRNA levels. Relative *DRAM* mRNA levels were expressed as ratios of normalized *DRAM* mRNA levels with Dox to those without Dox.

### Protein preparation and immunoblotting

Cells were harvested and resuspended in lysis buffer containing 50 mmol/L Tris-HCl (pH 8.0), 150 mmol/L NaCl, 5 mmol/L EDTA, and 1% protease inhibitor cocktail (Sigma-Aldrich). The lysate was analyzed by Western blotting as described previously [29] using anti-p53 (sc-6243; Santa Cruz Biotechnology, Santa Cruz, CA), anti-actin (A2066; Sigma-Aldrich), anti-LC3 (PM036; Medical & Biological Laboratories, Nagoya, Japan), anti-FLAG (M2: F3165; Sigma-Aldrich) antibodies. The intensity of LC3-I and LC3-II bands was measured by densitometry, and the LC3-II/I ratio was taken as a measure of the ability to induce autophagy.

### DRAM knockdown cell lines

DRAM expression was knocked down using the shRNA-mir GIPZ lentiviral vector (Thermo Fisher Scientific, Waltham, MA) targeting the sequence, 5′-CACAACACTATAAGAAATA-3′ in the 3′-UTR of *DRAM* mRNA. The sequence of nonsilencing control (NSC) was 5′-CTTACTCTCGCCCAAGCGAGAG-3′. p53-inducible cell lines (Saos-2Tet-p53-WT, Saos-2Tet-p53-S121F, SF126Tet-p53-WT, and SF126Tet-p53-S121F) were transduced with lentivirus targeting *DRAM* mRNA or targeting no mRNA (NSC). Cells were selected for stable integration of the lentivirus by incubation with 1 μg/mL puromycin (Wako, Osaka, Japan). All cells were examined under a fluorescence microscope BZ9000 (Keyence, Osaka, Japan) to detect green fluorescent protein (GFP) expression, which is an indicator of transduction efficiency.

### Flow cytometry

Cells were treated as described previously [29] and analyzed using the Cytomics FC500 flow cytometer (Beckman Coulter). At least 10,000 cells per sample were counted. The percentage of the subG1 fraction was taken as a measure of the ability to induce apoptosis.

### Statistical analysis

All experiments were repeated at least three times. The results are expressed as mean ± standard deviation (SD). The Student's *t*-test was performed and *P* < 0.05 was considered statistically significant.

## Results

### p53-inducible Saos-2 cells

**Figure 1:**
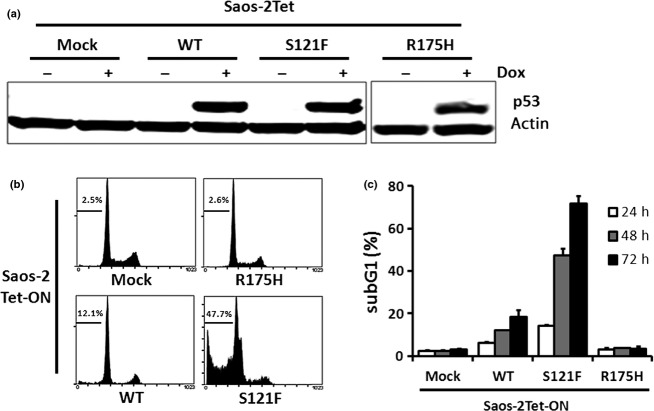
p53-inducible Saos-2 cells and ability to induce apoptosis. (a) p53 protein levels were detected by Western blotting 24 h after Dox treatment. (b) DNA histograms were analyzed by flow cytometry 48 h after Dox treatment. Horizontal bars and figures indicate the range of the subG1 fraction (%). (c) The subG1 fractions are presented as mean ± SD (%).

To confirm whether p53 was stably expressed in the established Dox-inducible clones, we performed Western blotting 24 h after Dox treatment and examined p53 protein levels. No significant change was observed in p53 protein levels of each clone (Fig. 1a). To evaluate the ability to induce apoptosis in each clone, we collected cultured cells at 24, 48, and 72 h after Dox treatment and analyzed the DNA histogram by flow cytometry. The subG1 fraction of wild-type p53 was significantly higher than that of Mock at 24, 48, and 72 h (*P* < 0.01 for all). Furthermore, the subG1 fraction of S121F was significantly higher than that of wild-type p53 at 24, 48, and 72 h (*P* < 0.01 for all). Apoptosis was not induced in R175H. These results indicate that S121F has an increased potent ability to induce apoptosis than wild-type p53 (Fig. 1b and c).

### S121F induces DRAM more strongly than wild-type p53

**Figure 2:**
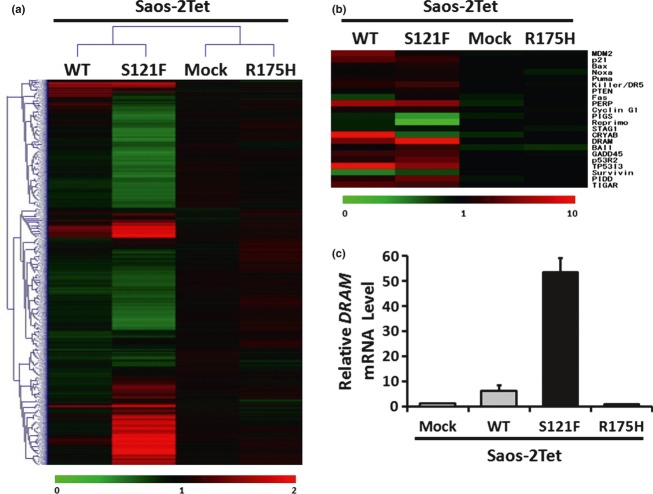
Identification of DRAM. (a) Microarray analysis was performed 24 h after Dox treatment. Hierarchical clustering of 22,111 genes is shown. The color gradation from green to red ranges from 0 to 2. (b) Twenty-two p53 downstream genes were selected from the microarray analysis data. The color gradation from green to red ranges from 0 to 10. (c) The *DRAM* mRNA levels were analyzed by qRT-PCR 24 h after Dox treatment.

To analyze the changes in the gene expression profile among Saos-2Tet-p53-WT, -S121F, and -R175H, we performed an oligonucleotide microarray analysis. Although more genes showed expression changes in S121F than in wild-type p53, the gene expression profile in S121F resembled that in wild-type p53. Few genes with expression changes were found in R175H (Fig. 2a). Among p53 downstream genes that were related to inhibition of cell proliferation, apoptosis, cell cycle arrest, and p53 degradation, we identified *DRAM* as one of the genes that were more upregulated by S121F than wild-type p53 (Fig. 2b). To confirm whether the *DRAM* mRNA level was more strongly induced by S121F than wild-type p53, we performed qRT-PCR. Similar to the results of microarray analysis, the *DRAM* mRNA levels were increased by wild-type p53 and S121F. Furthermore, *DRAM* was more significantly induced by S121F than wild-type p53 (*P* < 0.01), but was not induced by R175H (Fig. 2c). We could not detect DRAM protein by Western blotting or immunoprecipitation using commercial anti-DRAM antibodies.

### DRAM enhances wild-type p53-dependent apoptosis

**Figure 3:**
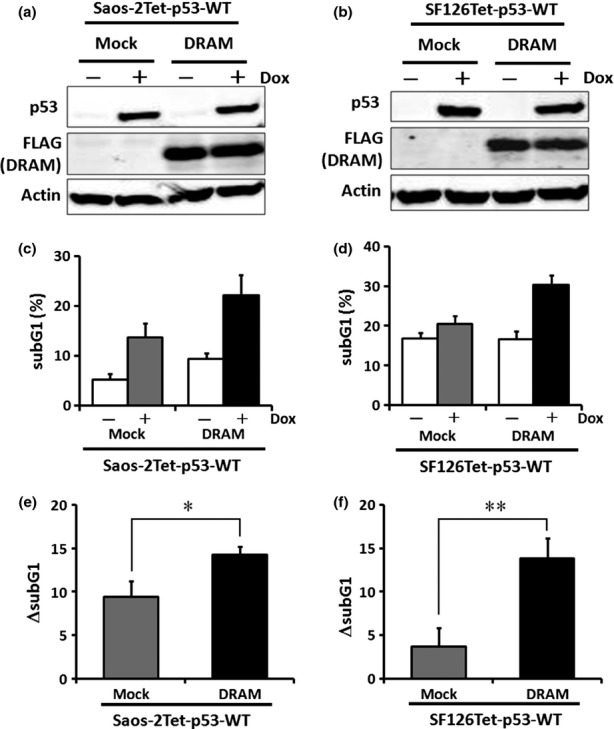
Ability of DRAM to induce apoptosis in the presence of wild-type p53. (a) and (b) Saos-2Tet-p53-WT or SF126Tet-p53-WT was transfected with plasmid encoding *DRAM*, and Western blotting was performed 24 h after Dox treatment. (c) and (d) Ability to induce apoptosis was analyzed by flow cytometry 48 h after Dox treatment. The subG1 fractions are presented as mean ± SD (%). (e) and (f) ΔsubG1 in (c) or (d) is presented as mean ± SD. *P < 0.05; **P < 0.01, by Student's *t*-test.

**Figure 4:**
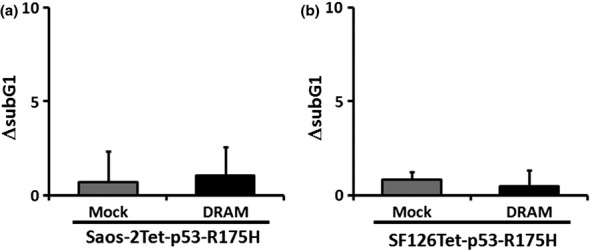
R175H does not induce apoptosis by DRAM overexpression. (a) and (b) Saos-2Tet-p53-R175H or SF126Tet-p53-R175H was transfected with plasmid encoding *DRAM* and their ability to induce apoptosis was analyzed by flow cytometry 48 h after Dox treatment. ΔsubG1 is presented as mean ± SD.

To analyze the ability of DRAM to induce apoptosis, we introduced a FLAG-tagged DRAM expression vector into Saos-2Tet-p53-WT or SF126Tet-p53-WT and performed Western blotting and flow cytometry. No distinct change was observed in p53 levels of each clone (Fig. 3a and b). In Saos-2 cells, the subG1 fractions of Mock and DRAM without wild-type p53 were 5.5 ± 1.6 and 9.7 ± 1.0, respectively, which showed that DRAM slightly induced apoptosis (*P* < 0.05) (Fig. 3c). In SF126 cells, the subG1 fractions of Mock and DRAM were 16.8 ± 1.6 and 16.5 ± 2.2, respectively, which were not significantly different (*P* = 0.87) (Fig. 3d). Next, we examined whether DRAM affected the ability to induce apoptosis in the presence of wild-type p53. To eliminate DRAM influence on apoptosis, we evaluated ΔsubG1, which was obtained by subtracting the value of the subG1 fraction without wild-type p53 from the value of the subG1 fraction with wild-type p53. Interestingly, in Saos-2 cells, although Mock ΔsubG1 was 9.4 ± 1.8 (mean ± SD), DRAM ΔsubG1 was significantly elevated up to 14.3 ± 1.0 (*P* < 0.05) (Fig. 3e). Similarly, in SF126 cells, although Mock ΔsubG1 was 3.7 ± 2.2, DRAM ΔsubG1 was significantly elevated up to 13.8 ± 2.3 (*P* < 0.01) (Fig. 3f). These results indicate that DRAM overexpression enhances wild-type p53-dependent apoptosis. Next, we examined whether DRAM affected the ability to induce apoptosis in the presence of R175H. No significant differences were observed in both Saos-2 and SF126 cells (*P* = 0.53 and *P* = 0.81, respectively) (Fig. 4a and b). These results indicate that DRAM enhances apoptosis in the presence of wild-type p53 but not in the presence of R175H. We also investigated the ability to induce autophagy in Saos-2Tet-p53-WT and SF126Tet-p53-WT. Both wild-type p53 alone and DRAM alone induced autophagy, and the ability of DRAM to induce autophagy was enhanced by wild-type p53 in SF126 cells, but not in Saos-2 cells (Fig. S1).

### Knockdown of DRAM does not affect the ability of S121F to induce apoptosis

**Figure 5:**
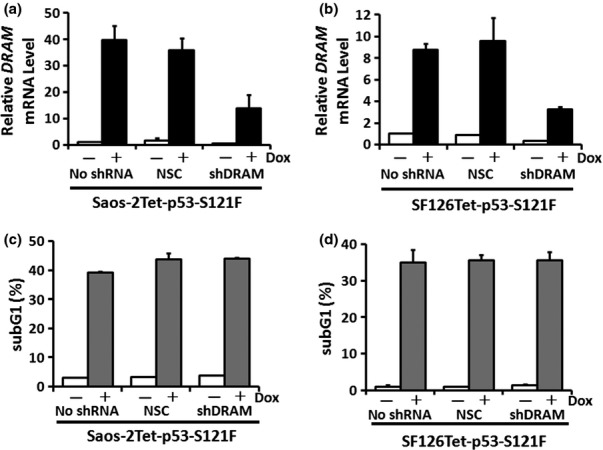
The influence of DRAM knockdown on the ability of S121F to induce apoptosis. (a) and (b) Saos-2Tet-p53-S121F or SF126Tet-p53-S121F was infected with lentivirus containing DRAM shRNA. The *DRAM* mRNA levels were analyzed by qRT-PCR 24 h after Dox treatment. Data are presented as the mean ± SD. NSC, nonsilencing control. (c) and (d) Ability to induce apoptosis was analyzed by flow cytometry 36 or 30 h after Dox treatment in (a) or (b), respectively. The subG1 fractions are presented as mean ± SD (%).

To analyze the role of DRAM to induce apoptosis and autophagy in S121F, we engineered endogenous DRAM knockdown system in Saos-2 and SF126 cell lines with Dox-inducible S121F using the lentivirus system. The cell lines infected with lentivirus containing *DRAM* shRNA were confirmed by fluorescence microscopy for GFP (data not shown). The *DRAM* mRNA levels were analyzed by qRT-PCR. We confirmed that the *DRAM* mRNA levels were downregulated in Saos-2Tet-p53-S121F-shDRAM and SF126Tet-p53-S121F-shDRAM compared with noninfected cells (no shRNA) or NSC (Fig. 5a and b). Using these DRAM knockdown cell lines, we examined whether DRAM knockdown affected the ability of S121F to induce apoptosis. No significant differences were observed in the ability to induce apoptosis in both Saos-2 and SF126 cells (*P* = 0.86 and *P* = 0.95, respectively) (Fig. 5c and d). We also investigated the ability to induce autophagy in each clone. S121F attenuated autophagy, but the ability to induce autophagy was not affected by DRAM knockdown (Fig. S2).

### Knockdown of DRAM attenuates the ability of wild-type p53 to induce autophagy but not apoptosis

**Figure 6:**
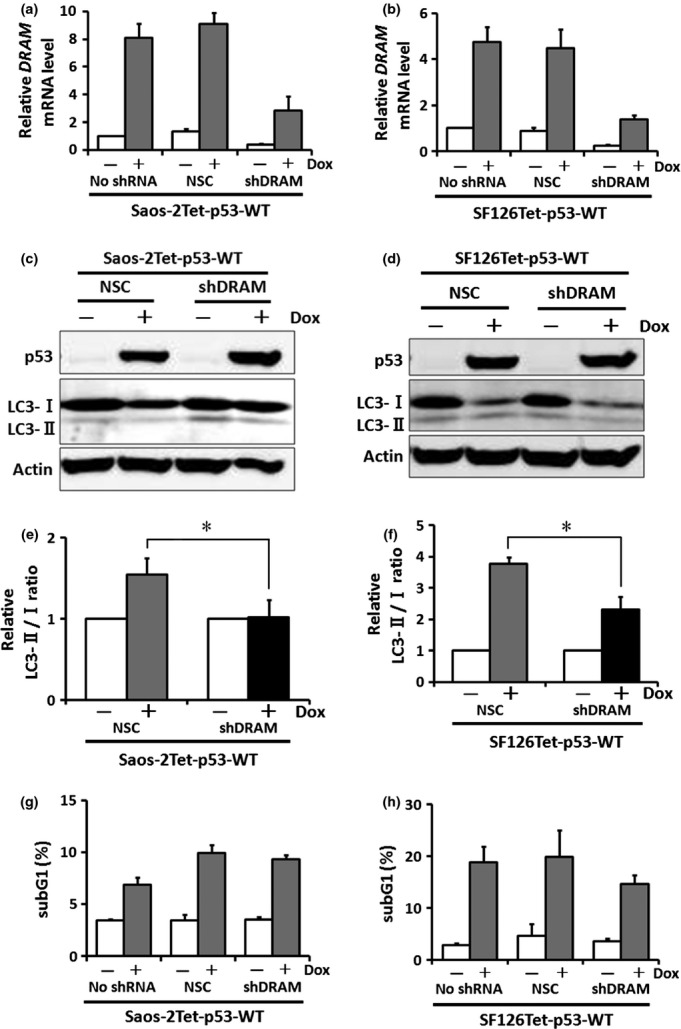
The influence of DRAM knockdown on the ability of wild-type p53 to induce apoptosis and autophagy. (a) and (b) Saos-2Tet-p53-WT or SF126Tet-p53-WT was infected with lentivirus containing *DRAM* shRNA. The *DRAM* mRNA levels were analyzed by qRT-PCR 24 h after Dox treatment. Data are presented as mean ± SD. NSC, nonsilencing control. (c) and (d) Expression levels of LC3-I and -II proteins were analyzed by Western blotting 24 h after Dox treatment. (e) and (f) Ability to induce autophagy was evaluated by the LC3-II/I ratio. Data are presented as mean ± SD. *P < 0.05 by Student's *t*-test. (g) and (h) Ability to induce apoptosis was analyzed by flow cytometry 48 h after Dox treatment. The subG1 fractions are presented as mean ± SD (%).

Next, we examined whether DRAM knockdown affected the ability to induce autophagy and apoptosis by wild-type p53. As previously mentioned, we established endogenous DRAM knockdown cell lines in wild-type p53. The *DRAM* mRNA levels were analyzed by qRT-PCR. We confirmed that the *DRAM* mRNA levels were downregulated in Saos-2Tet-p53-WT-shDRAM and SF126Tet-p53-WT-shDRAM compared with NSC clones (Fig. 6a and b). The relative values of LC3-II/I ratio in Saos-2Tet-p53-WT-NSC and SF126Tet-p53-WT-NSC in the presence of wild-type p53 were 1.6 ± 0.2 and 3.5 ± 0.5, respectively, and the ability to induce autophagy was increased in each clone. In Saos-2Tet-p53-WT-shDRAM and SF126Tet-p53-WT-shDRAM, the relative values of LC3-II/I ratio were 1.1 ± 0.2 and 2.0 ± 0.6, respectively. When compared with NSC clones, the relative values of LC3-II/I ratio were significantly decreased in shDRAM clones (*P* < 0.05 for both) (Fig. 6c–f). These results indicate that autophagy induced by wild-type p53 is attenuated by DRAM knockdown. Furthermore, as shown in Figure 6g and h, DRAM knockdown did not affect the ability of wild-type p53 to induce apoptosis in both Saos-2 and SF126 cells (*P* = 0.30 and *P* = 0.068, respectively).

## Discussion

In this study, we performed gene expression microarray analysis using a Dox-inducible p53 expression system to test the hypothesis that the mechanism of S121F-dependent apoptosis involves increased expression of genes of known or unknown functions.

First, the results suggested that DRAM was involved in the potent ability of S121F to induce apoptosis. *DRAM* has recently been reported as a p53 target gene and has been shown to be involved in apoptosis and autophagy induced by p53 [30].

Next, we showed that DRAM enhanced wild-type p53-dependent apoptosis. When DRAM alone was expressed, the influence on the ability to induce apoptosis was unchanged or minimal, but in the presence of wild-type p53, a significant enhancement was observed using flow cytometry. When wild-type p53 is expressed, the expression of the target gene *DRAM* increases and contributes to apoptosis induction, and more potent expression of *DRAM* is expected to further enhance wild-type p53-dependent apoptosis.

However, the mechanism of DRAM to enhance wild-type p53-dependent apoptosis remains unclear. One hypothesis is that DRAM localized to lysosomes acts in concert with the activation of the ability to induce apoptosis mediated by lysosome-dependent pathways resulting from p53 expression. Phosphorylated p53 is known to be localized in lysosomes and may contribute to enhanced apoptosis [31]. In addition, Bax, which is transcriptionally induced by activated p53, has been reported to be partially localized in lysosomes and to activate a lysosome-dependent apoptosis pathway [32]. Therefore, DRAM may enhance apoptosis induction via interaction with these proteins. Another hypothesis is that apoptosis and autophagy act in concert [33,34]. In DRAM-mediated apoptosis, Atg5, an important modulating factor in autophagy, is reported to play a role, and DRAM and Atg5 may be involved in some common pathways of apoptosis and autophagy [30]. Moreover, Sestrin1, Sestrin2, TSC2, AMPK, and PTEN, which are induced by wild-type p53, have also been shown to activate autophagy [35,36]. Constitutive factors in autophagy pathways including DRAM activated by wild-type p53 may act in concert with apoptotic factors. As shown in Figure S1, DRAM induced autophagy. Autophagy induced by DRAM expression may enhance the ability of wild-type p53 to induce apoptosis. Because our study shows the results of an overexpression experiment, further investigation is necessary to determine whether similar results can be obtained by activation of endogenous wild-type p53.

Although DRAM overexpression enhanced the ability of wild-type p53 to induce apoptosis and S121F induced *DRAM* expression more strongly than wild-type p53, no changes in the ability of S121F to induce apoptosis were noted when endogenous DRAM expression was suppressed. Several p53 target genes have been reported, and with regard to the transcription-dependent pathway, *Bax*,* Puma*,* Noxa*,* p53AIP1*, and *Killer/DR5* have been mentioned in the mechanism of p53-dependent apoptosis. In addition, when the transcription-independent pathway in which p53 directly acts in mitochondria and lysosomes to induce apoptosis is also included, apoptosis induction by p53 is expected to occur through multiple mechanisms. When the expression of one of the multiple p53 target genes is suppressed, there may be a compensatory increase in the expression of another target gene.

Crighton et al. reported that DRAM was critical for p53-dependent apoptosis [30]. In their experimental system, the ability of p53 to induce apoptosis was attenuated by DRAM knockdown. However, in our experimental system, the ability to induce wild-type p53-dependent apoptosis was not decreased by DRAM knockdown. For this reason, there is a possibility that DRAM knockdown was insufficient. For another reason, there is a possibility that DRAM is not necessary for apoptosis induction in the context of the experiment and type of cells. Further experimentation will be necessary.

About half of all human cancers retain wild-type p53, so that by further increasing *DRAM* expression, the antitumor activity of wild-type p53 may be enhanced. In other words, if DRAM expression levels in cancer tissues can be increased by stimulating *DRAM* transcription or inhibiting DRAM degradation, the therapeutic effects of anticancer drugs and radiation therapy may be further enhanced.

## Supporting Information

Figure S1. Ability of DRAM to induce autophagy.

Figure S2. Ability of S121F to induce autophagy.
