# Supplementary figures and images for: Overexpression of DRAM enhances p53-dependent apoptosis
Source: Cancer Med. 2013 Feb 3;2(1):1–10. doi: 10.1002/cam4.39 (PMC3797565; doi:10.1002/cam4.39)

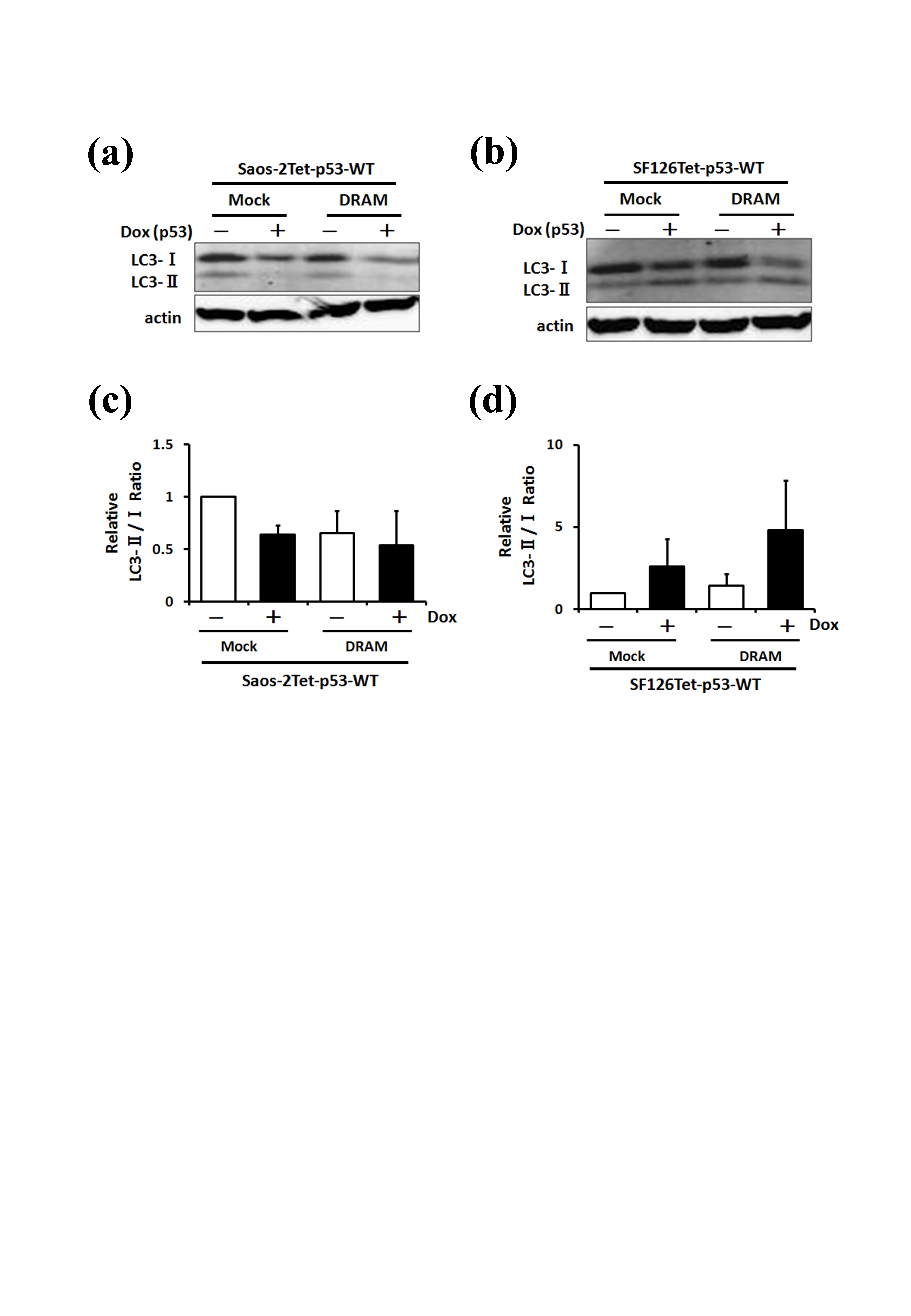

Supplement: Figure S1. — Ability of DRAM to induce autophagy. [file cam0002-0001-SD1.tif]

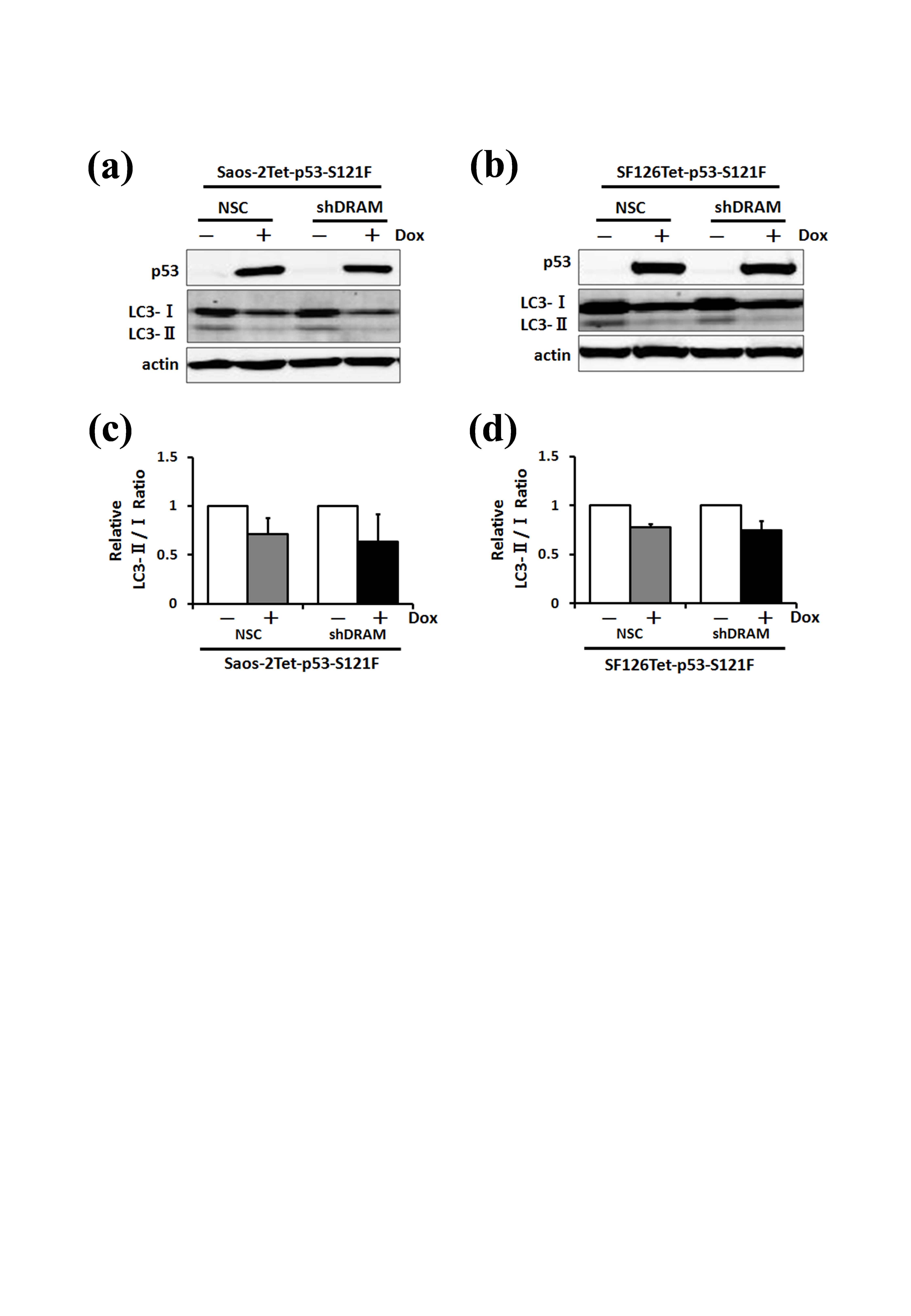

Supplement: Figure S2. — Ability of S121F to induce autophagy. [file cam0002-0001-SD2.tif]
